# Bispecific Antibodies for Acute Myeloid Leukemia: From Bone Marrow Immune Niche to Clinical Translation

**DOI:** 10.3390/antib15040069

**Published:** 2026-08-04

**Authors:** Antonella Bruzzese, Enrica Antonia Martino, Santino Caserta, Maria Eugenia Alvaro, Nicola Amodio, Eugenio Lucia, Virginia Olivito, Caterina Labanca, Francesco Mendicino, Fortunato Morabito, Ernesto Vigna, Massimo Gentile

**Affiliations:** 1 Hematology Unit, Department of Onco-Hematology, AO of Cosenza, Viale della Repubblica snc, 87100 Cosenza, Italy; a.bruzzese@aocs.it (A.B.); enricaantoniamartino@gmail.com (E.A.M.); s.caserta@aocs.it (S.C.); m.eugenia983@gmail.com (M.E.A.); e.lucia@aocs.it (E.L.); v.olivito@aocs.it (V.O.); c.labanca@aocs.it (C.L.); f.mendicino@aocs.it (F.M.); ernesto.vigna@aocs.it (E.V.); 2 Department of Experimental and Clinical Medicine, University of Catanzaro, 88100 Catanzaro, Italy; amodio@unicz.it; 3 Department of Pharmacy, Health and Nutritional Science, University of Calabria, 87036 Rende, Italy; f.morabito53@gmail.com; 4 AIL Sezione di Cosenza, 87100 Cosenza, Italy

**Keywords:** acute myeloid leukemia, bispecific antibodies, leukemic stem cells, bone marrow microenvironment, immunotherapy

## Abstract

Acute myeloid leukemia (AML) is a heterogeneous hematologic malignancy characterized by the clonal expansion of myeloid blasts and the persistence of leukemic stem cells (LSCs) within a profoundly remodeled bone marrow (BM) microenvironment. Despite advances in molecular stratification and the introduction of targeted agents, long-term outcomes remain unsatisfactory, particularly in older and high-risk patients. Increasing evidence indicates that leukemogenesis and treatment resistance are critically sustained by a permissive immune milieu, in which LSCs, myeloid-derived suppressor cells, leukemia-associated macrophages, and dysfunctional T and NK cells shape an immunosuppressive “leukemic niche.” This evolving understanding has renewed interest in immune-based strategies capable of restoring effective antitumor immunity. Bispecific antibodies (bsAbs) are engineered molecules designed to engage AML-associated antigens while simultaneously recruiting and activating immune effector cells, most commonly T cells or NK cells. By promoting immune synapse formation independently of major histocompatibility complex expression and conventional co-stimulatory pathways, bsAbs can overcome several mechanisms of immune escape. In this review, we summarize the biological rationale for immunotherapy in AML, with a focus on the role of the BM microenvironment and immune dysregulation. We then discuss the structural and functional properties of IgG-like and non-IgG-like bsAbs, key antigenic targets such as CD33, CD123, CD70 and others, and the main T-cell- and NK-cell-engaging platforms under clinical investigation. Finally, we highlight emerging clinical data, principal toxicities, and the challenges of integrating bsAbs into existing treatment algorithms, including combinations with hypomethylating agents, BCL-2 inhibitors, and allogeneic stem cell transplantation. A deeper understanding of AML immune biology and antigen expression patterns will be essential to optimize bsAb design, maximize therapeutic benefit, and minimize on-target off-tumor toxicity.

## 1. Introduction

Acute myeloid leukemia (AML) comprises a heterogeneous group of aggressive hematologic malignancies characterized by the uncontrolled proliferation and accumulation of myeloid blasts [1,2,3,4,5]. Despite extensive efforts to elucidate the mechanisms underlying leukemogenesis, the etiology of AML is incompletely understood and likely results from a complex interplay of genetic, epigenetic, and environmental factors [1,2,3,4,5].

Advances in the understanding of these pathogenic mechanisms have led to the development of several targeted therapies that have recently been incorporated into AML treatment strategies. Consequently, current therapeutic approaches are determined not only by patient fitness, but also by the disease’s biological and molecular features [3,4,6].

Recent AML classification systems, including those proposed by the International Consensus Classification (ICC) and the World Health Organization (WHO), increasingly emphasize the molecular landscape of the disease, refining AML subtypes and incorporating novel genetic entities into the classification of myeloid neoplasms [3,4]. To date, more than 5000 driver mutations have been identified in AML, involving genes associated with RNA splicing, chromatin remodeling, cellular differentiation, cell-cycle regulation, and growth factor signaling pathways [7,8]. Importantly, the prognostic impact of individual mutations is often influenced by the presence of co-occurring genetic alterations, highlighting the remarkable biological complexity of AML pathogenesis [9]. However, genetic lesions alone do not fully explain treatment resistance and disease persistence, underscoring the importance of the bone marrow (BM) microenvironment and immune dysregulation in AML pathogenesis.

In addition to molecular and cytogenetic abnormalities arising in hematopoietic progenitors, increasing evidence suggests that the BM microenvironment also plays a crucial role in AML initiation and progression. Emerging data support the existence of a dynamic and complex crosstalk between AML cells and the BM microenvironment, which contributes to the establishment of a permissive immune milieu capable of protecting leukemic stem cells (LSCs) from therapy-induced apoptosis and thereby promoting disease persistence. The identification of immune targets expressed by AML cells, together with strategies aimed at modulating the BM microenvironment, may enhance the anti-leukemic activity of current therapies, restore immune surveillance, and ultimately improve clinical outcomes.

### 1.1. Role of Immune Response in AML

The hierarchical organization of leukemic cells originates from LSCs, which are characterized by their self-renewal capacity and their ability to initiate leukemia following transplantation into SCID mice [10,11].

Although LSCs are known to be enriched within the CD34+ CD38- cell fraction, a definitive phenotypic characterization of these cells has not yet been established. Compared with normal hematopoietic stem cells (HSCs), LSCs have been reported to exhibit increased expression of several surface markers, including CD25, CD32, CD44, CD96, CD123, CD200, CCL-1, c-MPL, GPR56, HDM2, N-cadherin, Tie2, and TIM-3 [12,13,14,15,16,17,18,19,20]. Numerous studies have demonstrated the adverse prognostic impact of LSC burden both at diagnosis and after treatment, leading to the development of several prognostic scoring systems based on gene expression profiling and whole-exome sequencing analyses [21].

The detrimental role of LSCs is largely related to their ability to reside within the BM niche in a quiescent state. This process progressively reshapes the physiological BM microenvironment into a “leukemic niche” capable of supporting leukemic cell proliferation and survival while impairing normal hematopoiesis. Furthermore, localization within the BM niche enables LSCs to evade both antileukemic therapies and immune-mediated cytotoxicity [22,23,24].

LSCs also contribute to the establishment of an immunosuppressive BM microenvironment. Two major immunosuppressive cellular populations have been identified: myeloid-derived suppressor cells (MDSCs) and leukemia-associated macrophages (LAMs). MDSCs represent a heterogeneous population of immature myeloid cells able to induce T-cell tolerance through multiple mechanisms. These cells express high levels of immune inhibitory molecules, including PD-L1, V-domain Ig suppressor of T-cell activation (VISTA), indoleamine 2,3-dioxygenase (IDO), arginase, reactive oxygen species (ROS), transforming growth factor-β (TGF-β), and interleukin-10 (IL-10) [25,26,27]. Given their role in promoting immune evasion and leukemic cell survival, elevated MDSC levels have been associated with poor clinical outcomes [28,29].

LAMs are macrophages representing critical components of the BM microenvironment and are generally characterized by an M2-like, pro-tumoral phenotype. However, the mechanisms through which the leukemic microenvironment induces macrophage polarization into LAMs remain largely unclear [30]. Collectively, these alterations generate a profoundly immunosuppressive leukemic niche that not only supports LSC maintenance but also represents an attractive target for novel immunotherapeutic strategies.

Other environmental cells contributing to leukemogenesis are CXCL12-abundant reticular (CAR) cells and mesenchymal stem cells (MSCs), key stromal components of the bone marrow niche that play a central role in shaping the AML microenvironment. CAR cells, a specialized subset of perivascular MSCs, produce high levels of stem cell factors (SCF) and CXCL12 (SDF-1), which are essential for hematopoietic stem cell maintenance under physiological conditions. In AML, the activation of the CXCL12/CXCR4 axis hijacks LSCs in the protective niches, where they receive survival, quiescence, and anti-apoptotic signals that promote chemotherapy resistance. Likewise, MSCs actively support AML progression through direct cell–cell interactions and the secretion of chemokines, cytokines, and extracellular matrix components able to suppress apoptosis, enhance neoplastic cells’ proliferation, and remodel the immune response. AML-associated MSCs also acquire an altered phenotype characterized by increased immunosuppressive activity and the production of soluble mediators that impair normal hematopoiesis while fostering leukemic persistence [23,24,31].

The increasing understanding of the BM microenvironment, together with its dynamic remodeling into a leukemic “sanctuary,” has profoundly changed the traditional view of leukemogenesis as merely the consequence of accumulating genetic abnormalities within leukemic cells. AML pathogenesis is now recognized as strongly dependent on the active bidirectional crosstalk between leukemic blasts and the BM niche.

Within this context, several emerging therapeutic strategies aim to restore immune surveillance within the BM microenvironment by targeting leukemic cell adhesion to the BM niche, reversing BM remodeling processes, disrupting vascular support, and modulating metabolic pathways involved in drug resistance [32].

In this complex interplay between leukemic cells and the pro-leukemic BM microenvironment, immune cells play a central role. In healthy BM, T cells contribute to hematopoietic regulation through chemokine receptor expression and cytokine secretion, whereas natural killer (NK) cells exert potent antitumor activity. Moreover, the BM represents the primary reservoir of B-cell precursors derived from HSCs, as well as long-lived plasma cells contributing to long-term humoral immunity [33,34,35,36].

Beyond remodeling the stromal niche, AML cells can also develop multiple strategies to evade immune surveillance by directly impairing the function of immune effector cells and preventing immune-mediated eradication. AML cells may undergo immune-editing processes that reduce immune recognition through epigenetic downregulation or loss of HLA molecules, increased expression of inhibitory ligands and immune checkpoint molecules, and enhanced secretion of immunosuppressive cytokines. Furthermore, AML cells promote the expansion of regulatory T cells (Tregs) and MDSCs and drive macrophage polarization toward an M2-like phenotype, further amplifying immune tolerance and treatment resistance [37,38,39].

Several studies have demonstrated substantial interpatient variability in the BM immune landscape of AML patients. In particular, immune profiles characterized by increased frequencies of CD8+PD-1+ T cells, enrichment of TH17/Treg intermediate populations, CD8+ memory T cells, dysfunctional macrophages, and specific dendritic cell subsets have been associated with poor prognosis [40,41].

**Figure 1:**
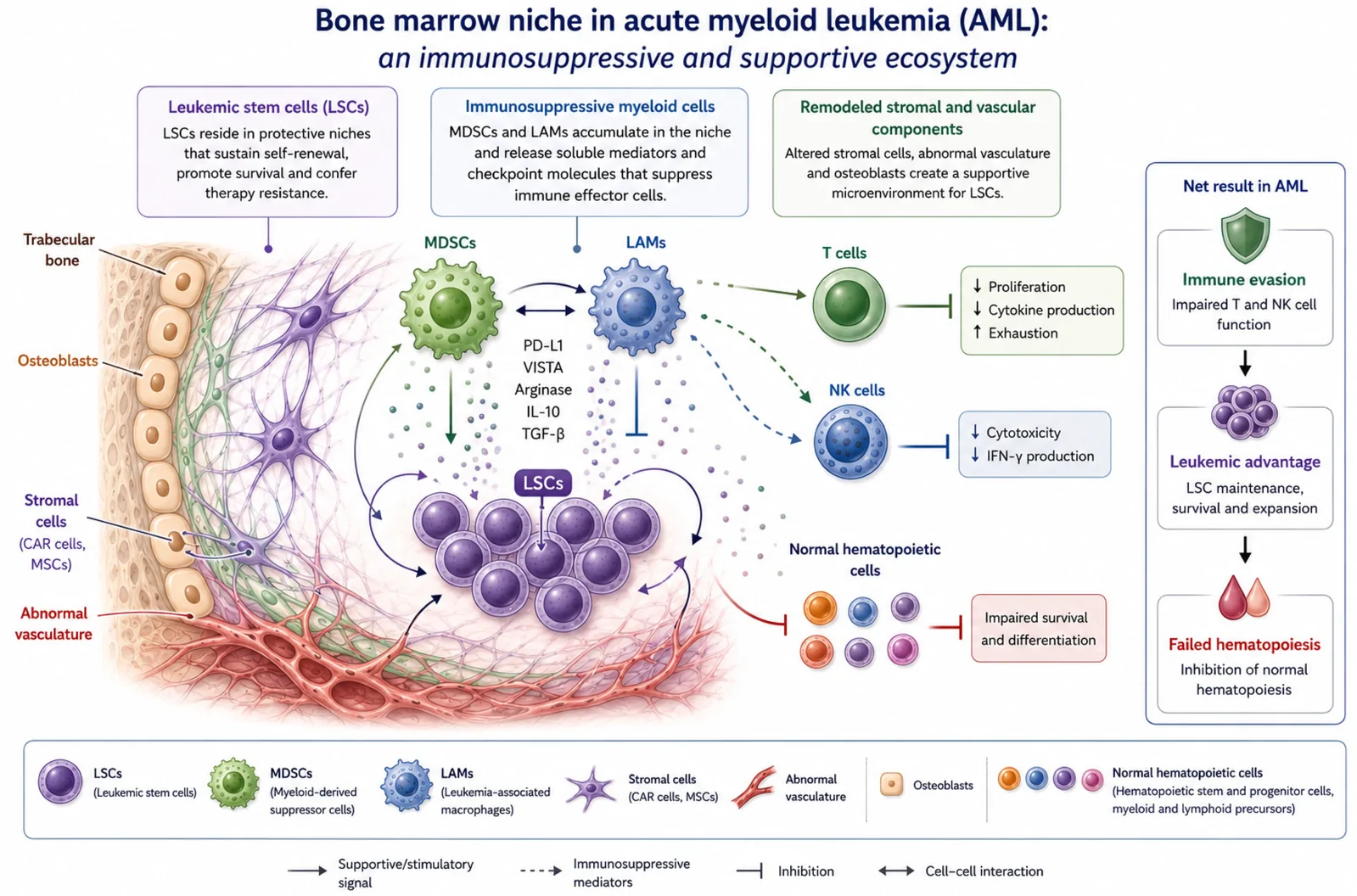
**Schematic representation of the leukemic bone marrow niche in AML.** Leukemic stem cells (LSCs) localize within a remodeled stromal microenvironment characterized by myeloid-derived suppressor cells (MDSCs), leukemia-associated macrophages (LAMs), and altered stromal and vascular elements. These components collectively secrete immunosuppressive mediators (e.g., PD-L1, VISTA, arginase, IL-10, TGF-β) that impair T- and NK-cell function, support LSC survival, and inhibit normal hematopoiesis. Partially prepared with the aid of an AI-based illustration tool and then edited by the authors.

The BM microenvironment in AML is schematically represented in Figure 1.

Over the last few decades, the remarkable clinical efficacy of immunotherapeutic approaches in lymphoid malignancies has stimulated the development of multiple immune-based strategies for AML treatment. However, major challenges remain, including the identification of optimal target antigens in the context of the marked biological heterogeneity of AML, the relatively low antigen density on AML cells compared with lymphoid malignancies, the difficulty in identifying antigens selectively expressed on LSCs, and the choice between antibody-based and cell-based therapeutic approaches.

### 1.2. Target for Immunotherapy in AML

Monoclonal antibodies (mAbs) have been widely developed and successfully implemented across several malignancies, particularly in B-cell neoplasms. In contrast, translating these successes to myeloid malignancies has proven more challenging because leukemic blasts and normal myeloid precursors share many surface antigens, raising concerns about prolonged cytopenias and marrow aplasia. The ideal targets of mAbs are lineage-specific antigens (LSAs) expressed at distinct stages of hematopoietic differentiation. Consequently, in myeloid malignancies such as AML, leukemic cells and normal myeloid precursors share most surface antigens, complicating target selection and increasing the risk of off-tumor toxicity and prolonged cytopenias.

In AML therapeutics, several mAbs have been developed against surface antigens that are overexpressed on leukemic cells and involved in survival and immune evasion pathways, including CD33, CD123, CD25, CD44, CD47, and CLL-1. These antibodies exert their effects through direct mechanisms, such as inhibition of proliferative signaling, interference with tumor cell survival, and blockade of ligand–receptor interactions, as well as through immune-mediated effector functions. Indirect mechanisms include activation of antibody-dependent cellular phagocytosis (ADCP) and antibody-dependent cell-mediated cytotoxicity (ADCC) [42,43,44].

#### 1.2.1. CD33

One of the earliest and most validated targets in AML immunotherapy is CD33, due to its relatively low expression on normal HSCs and mature granulocytes compared with its high expression on AML blasts. Accordingly, CD33 has emerged as a key therapeutic target in AML [45]. Gemtuzumab ozogamicin (GO) was the first humanized anti-CD33 antibody–drug conjugate approved by the Food and Drug Administration (FDA) and the European Medicines Agency (EMA). GO, conjugated with calicheamicin, has demonstrated improved outcomes in patients with standard- and intermediate-risk AML when combined with chemotherapy. In the phase III ALFA-0701 trial, the addition of GO to standard chemotherapy resulted in a 3-year event-free survival (EFS) of 40.8% versus 17.1% with chemotherapy alone (HR 0.58; *p* = 0.0003) in de novo AML patients, with complete remission (CR) rates of 81% versus 75%, respectively [46].

#### 1.2.2. CD45

Another myeloid antigen explored as a therapeutic target is CD45, a type I transmembrane protein expressed as multiple isoforms on nearly all hematopoietic cells, except erythrocytes and platelets [47,48]. To date, the main therapeutic approach targeting CD45 has involved radiolabeled mAbs. In a phase I study, yttrium-90-labeled anti-CD45 antibody (90Y-DOTA-BC8) was evaluated in patients with high-risk AML, acute lymphoblastic leukemia (ALL), or myelodysplastic syndromes (MDS), demonstrating that its combination with allogeneic stem cell transplantation (alloSCT) was safe and associated with improved post-transplant outcomes [49].

#### 1.2.3. CD47

CD47, which is broadly expressed on myeloid leukemia cells, functions as a “don’t eat me” signal through its interaction with signal regulatory protein α (SIRPα), thereby inhibiting macrophage-mediated phagocytosis. In AML, CD47 overexpression correlates with poor overall survival and is particularly enriched on LSCs, contributing to immune evasion. Preclinical studies have shown that anti-CD47 mAbs enhance phagocytosis of LSCs and prevent AML engraftment [50]. The anti-CD47 antibody CC-90002 disrupts the CD47–SIRPα axis and demonstrates significant antitumor activity in murine models. However, a phase I clinical trial evaluating CC-90002 monotherapy in relapsed/refractory (R/R) AML or high-risk MDS did not confirm these preclinical results [51,52]. Similarly, magrolimab initially showed promising activity in combination with azacitidine, achieving CR/CR with incomplete recovery (CRi) rates of 64% in treatment-naïve AML patients with a median overall survival of 18.9 months. Nevertheless, enthusiasm declined following the phase III ENHANCE-2 trial, which failed to demonstrate an overall survival benefit compared with venetoclax plus azacitidine in TP53-mutated AML and was terminated early [53,54]. Ligufalimab (AK117), a novel humanized anti-CD47 IgG4 antibody lacking hemagglutination activity, has demonstrated a favorable safety profile and is currently under clinical evaluation [55]. These findings illustrate both the strong biological rationale for targeting CD47 and the challenges of translating this approach into durable clinical benefit in unselected AML populations.

#### 1.2.4. CD70

CD70, a member of the tumor necrosis factor (TNF) family, has also emerged as a promising target in AML because it is selectively expressed on leukemic cells and largely absence from normal hematopoietic stem cells. Cusatuzumab (ARGX-110) is a mAb with an engineered Fc region targeting CD70 and mediating leukemic cell killing through antibody-dependent cellular cytotoxicity (ADCC) [56,57]. Preclinical and clinical data suggest that azacitidine may represent an optimal partner for ARGX-110, as it upregulates CD70 expression on LSCs. Additional studies indicate that combining cusatuzumab with venetoclax may further enhance its antileukemic activity [58,59,60]. Ongoing clinical trials are evaluating cusatuzumab in combination with azacitidine and venetoclax (NCT06384261). These combinations exemplify a broader strategy of integrating targeted immunotherapies with venetoclax- and HMA-based backbones that have become standard in older or unfit AML patients.

#### 1.2.5. CD123

Compared with normal hematopoietic stem cells, AML blasts overexpress CD123, the α-chain of the interleukin-3 receptor (IL-3Rα). Several therapeutic agents targeting CD123 have been investigated with variable clinical success [61,62,63,64]. Among them, particular attention has been given to tagraxofusp, owing to its established efficacy in blastic plasmacytoid dendritic cell neoplasm (BPDCN) [65]. Following these results, multiple clinical trials have evaluated its activity in AML and other myeloid malignancies [61,66].

### 1.3. Rationale for Bispecific Antibodies in AML

Considering the profound immune evasion mechanisms described above, conventional mAbs and antibody–drug conjugates have provided only modest and often transient benefit in AML. Shared expression of target antigens between leukemic cells and normal hematopoietic progenitors leads to on-target off-tumor toxicity, whereas the relatively low antigen density on AML blasts and LSCs may limit the potency of single-antigen approaches. In addition, the logistical and toxicity challenges of cellular immunotherapies, such as chimeric antigen receptor (CAR) T cells, have thus far hampered their broad implementation in AML. Bispecific antibodies (bsAbs) offer a potentially attractive alternative by enabling off-the-shelf immune redirection against AML and LSCs, while allowing flexible targeting strategies that may mitigate toxicity and overcome immune escape.

### 1.4. Bispecific Antibodies

BsAbs are engineered molecules capable of simultaneously recognizing two distinct antigens, thereby enabling targeting strategies that are not achievable with conventional immunoglobulin G (IgG)-based mAbs. Structurally, bsAbs are commonly classified into two main categories: IgG-like and non-IgG-like molecules.

IgG-like bsAbs retain the Fc region, which facilitates purification and improves solubility and structural stability. In addition, the Fc domain can mediate effector functions such as antibody-dependent cellular cytotoxicity (ADCC), complement-dependent cytotoxicity (CDC), and antibody-dependent cellular phagocytosis (ADCP). Owing to their larger molecular size and FcRn-mediated recycling, IgG-like bsAbs typically exhibit prolonged serum half-lives; however, their tissue penetration is relatively limited.

In contrast, non-IgG-like bsAbs lack the Fc region and therefore display several advantages, including improved tissue penetration, simpler manufacturing processes, and the ability to engage epitopes that may be sterically inaccessible in full-length IgG formats. Moreover, the absence of an Fc domain may reduce immunogenicity. However, these formats, particularly single-chain variable fragment (scFv)-based constructs, are generally characterized by short plasma half-lives, rapid systemic clearance, fast dissociation rates, and limited retention at target sites. Consequently, multiple engineering and formulation strategies have been developed to improve their bioavailability and enhance therapeutic efficacy [66]. In practice, the optimal bsAb format in AML must balance sufficient half-life and exposure with adequate bone marrow and tissue penetration, while minimizing off-target engagement and cytokine-mediated toxicity.

Functionally, bsAbs exert their activity through dual antigen specificity, typically recognizing a tumor-associated antigen and a surface antigen expressed on immune effector cells such as T cells or natural killer (NK) cells. This dual engagement brings malignant cells into proximity with effector cells, promoting immune activation and resulting in enhanced cytotoxic killing of tumor cells. Importantly, this mechanism is largely independent of major histocompatibility complex (MHC) expression and costimulatory molecules on tumor cells, thereby overcoming immune evasion strategies such as downregulation of these pathways on the tumor cell surface [67,68,69,70].

**Figure 2:**
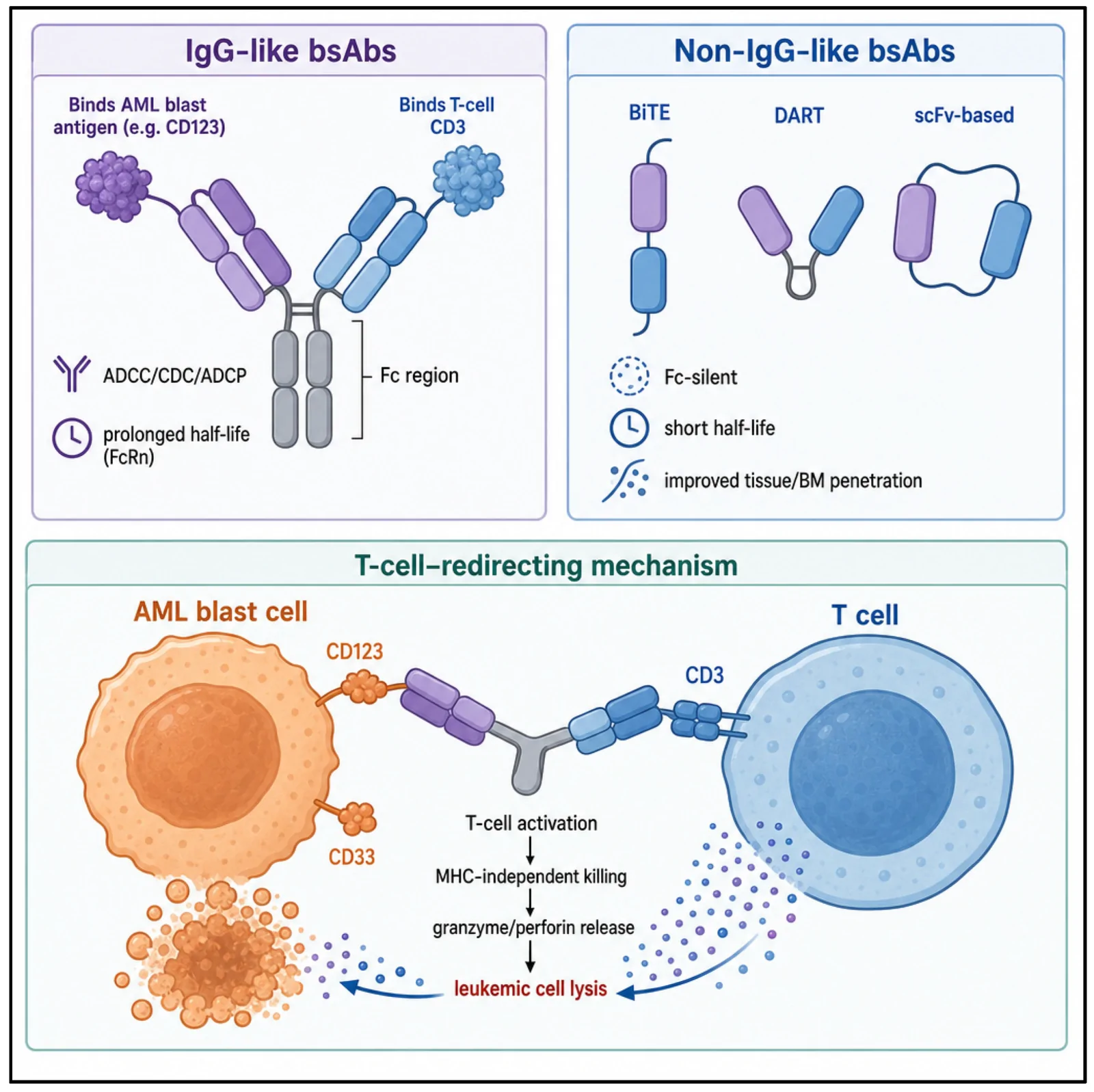
**Bispecific antibody formats and mechanisms of action.** Schematic overview of the main bispecific antibody (bsAb) formats and their T-cell-redirecting mechanism in acute myeloid leukemia (AML). The left panel illustrates IgG-like bsAbs, which retain an Fc region conferring structural stability, Fc-mediated effector functions (antibody-dependent cellular cytotoxicity, complement-dependent cytotoxicity, and antibody-dependent cellular phagocytosis), and prolonged serum half-life through FcRn-mediated recycling, at the cost of relatively limited tissue and bone marrow penetration. The right panel shows non-IgG-like bsAbs, such as BiTE- or DART-like single-chain variable fragment-based constructs, which lack an Fc domain and therefore exhibit shorter half-lives but improved tissue and bone marrow penetration and simplified manufacturing. The lower panel depicts the T-cell-redirecting mechanism common to many bsAbs: simultaneous engagement of a tumor-associated antigen (e.g., CD33 or CD123) on AML blasts and CD3 on T cells leads to immune synapse formation, T-cell activation and proliferation, and MHC-independent release of cytotoxic granules (perforin and granzymes), ultimately resulting in leukemic cell lysis. Partially prepared with the aid of an AI-based illustration tool and then edited by the authors.

Based on their mechanisms of action, bsAbs can be further categorized into three functional groups: (i) molecules that bridge immune and tumor cells to recruit and activate effector cells for tumor killing, (ii) agents that simultaneously modulate multiple signaling pathways to generate synergistic effects, and (iii) constructs that facilitate the formation of protein complexes to induce specific biological activities [71]. The main structural formats of bsAbs and their T-cell-redirecting mechanism of action are summarized in Figure 2.

Bispecific T-cell engagers (BiTEs) represent the first clinically successful class of bsAbs in hematological malignancies. They function by recruiting cytotoxic T cells to tumor cells through simultaneous binding to CD3 on T cells and tumor-associated antigens on malignant cells. This interaction triggers T-cell activation independently of classical costimulatory signals such as interleukin-2 (IL-2) and CD28, thereby maintaining activity even in conditions of impaired immune function or reduced costimulatory signaling. This property underlies the broad clinical application of BiTEs in hematologic malignancies [68].

In acute myeloid leukemia (AML), multiple bsAbs are currently under clinical investigation, with several leukemic surface antigens emerging as promising therapeutic targets.

#### 1.4.1. Anti-CD123 Bispecific Antibodies

The α-chain of the interleukin-3 receptor, also known as CD123, is frequently overexpressed on leukemic blast cells and is associated with increased proliferative capacity and resistance to apoptosis, correlating with poor clinical outcomes. Several therapeutic agents targeting CD123 have therefore been evaluated in AML [61,62,63,64,65,66].

APVO436 is a recombinant IgG-like Fc-containing bispecific antibody designed to redirect host T-cell cytotoxicity toward CD123-expressing blasts in an MHC-independent manner. APVO436 induces T-cell recruitment, activation, and proliferation with limited cytokine release, resulting in concentration-dependent lysis of CD123-positive leukemic cells.

In preclinical mouse models, co-administration of human T cells and APVO436 demonstrated potent, dose-dependent anti-leukemic activity, significantly improving survival in a NOD/SCID xenograft model of human AML. Efficacy was observed at doses ≥0.02 μg/mouse (approximately 1 μg/kg; human equivalent dose [HED] 0.08 μg/kg), with maximal activity at an HED of 0.4 μg/kg. In cynomolgus monkey studies, single intravenous doses ranging from 0.25 to 1 mg/kg were well tolerated. Repeated dosing over four weeks at 0.5, 2.5, and 10 mg/kg per dose also showed no clinical, laboratory, or histopathological evidence of systemic toxicity or organ damage [69,70,71].

A first-in-human phase Ib clinical trial evaluated APVO436 in R/R AML and high-risk MDS. A total of 46 patients, previously treated with 1–8 prior lines of therapy, received weekly infusions across 10 dose levels (0.3–60 μg). APVO436 demonstrated a favorable safety profile with manageable treatment-related adverse events (TRAEs), and the maximum tolerated dose (MTD) was not reached at 60 μg weekly. The most common TRAEs were infusion-related reactions (28.3%) and cytokine release syndrome (CRS) (21.7%). The recommended phase 2 dose (RP2D) was identified at 0.2 μg/kg. Regarding efficacy, 3 of 6 evaluable High-risk MDS patients achieved marrow complete remission, while partial responses, complete remissions, and prolonged stable disease were also observed in R/R AML patients at the RP2D [72].

A post hoc analysis of CRS-related outcomes showed that 4 patients developed grade ≥3 CRS. No clear association was identified between CRS occurrence and baseline clinical variables such as disease burden, hematologic parameters, or demographic factors, except for an age difference (median of 73.5 vs. 65 years; *p* = 0.04). Premedication with steroids did not eliminate CRS risk. Cytokine profiling revealed a predominance of IL-6 in patients with CRS. CRS events were generally manageable with tocilizumab, with or without dexamethasone, and importantly, CRS occurrence was not associated with treatment response, as clinical benefit was observed in both CRS and non-CRS patients [73].

Vibecotamab (XmAb14045), a humanized bispecific antibody targeting CD3 and CD123, promotes T-cell recruitment and activation leading to selective killing of CD123-positive leukemic cells. A phase Ib study in R/R AML implemented step-up dosing to reduce CRS incidence, followed by weekly administration (1.7 μg/kg). DLTs were observed in 13 of 120 patients (16%), with CRS being the most frequent TRAE (59.2%), predominantly grade ≤2. The overall response rate was 9.0%, with responses primarily observed at doses ≥0.75 μg/kg and associated with lower baseline blast counts in peripheral blood and BM (<25%) [74].

Flotetuzumab (MGD006/S80880), a dual-affinity retargeting (DART) molecule, consists of two polypeptide chains, each containing the variable heavy (VH) domain of one antibody fused in tandem with the variable light (VL) domain of the other. Flotetuzumab simultaneously binds CD3 and CD123, promoting immune synapse formation, T-cell activation, proliferation, and receptor diversification, resulting in dose-dependent cytotoxicity against AML cell lines and primary blasts in vitro and in vivo [75].

In a phase I/II clinical study involving 88 adults with R/R AML, flotetuzumab demonstrated an overall response rate of 30% in primary refractory or early relapsed patients, with a complete remission/CR with partial hematologic recovery (CR/CRh) rate of 26.7%. Among responders, median overall survival was 10.2 months, with 6- and 12-month survival rates of 75% and 50%, respectively. Transcriptomic analyses identified a 10-gene signature predictive of response. Notably, patients with TP53-mutated AML achieved complete responses in 47% of cases, accompanied by a more inflamed tumor microenvironment characterized by higher CD8, FOXP3, and PD-1 gene expression. These findings suggest that flotetuzumab, acting mostly with the activation of microenvironment irrespective of leukemic cells metabolism, may overcome resistance associated with adverse molecular features [76,77].

JNJ-63709178, another dual-targeting antibody, was evaluated in a phase I study using both intravenous and subcutaneous step-up dosing regimens. High rates of grade ≥3 treatment-related adverse events were observed (65% in early cohorts and 92% in later cohorts). At the highest intravenous dose (4.8 μg/kg), 71% of patients discontinued treatment due to toxicity. Both intravenous and subcutaneous administration were associated with cytokine release and infusion-related reactions, and step-up dosing did not substantially improve safety. Clinical efficacy was limited [78].

#### 1.4.2. Anti-CD33 Bispecific Antibodies (bsAbs)

JNJ-67571244 is a CD33 × CD3 bispecific antibody that binds the CD33 C2 domain on AML blasts and CD3 on T cells, thereby inducing T-cell activation and redirecting cytotoxic T lymphocytes toward CD33-expressing leukemic cells. In preclinical studies, JNJ-67571244 demonstrated selective binding to CD33-positive AML cells, potent in vitro T-cell-dependent cytotoxicity, and significant in vivo antitumor activity. Its safety and efficacy have recently been evaluated in patients with R/R AML or high-risk MDS in an open-label phase I study. The drug was administered intravenously or subcutaneously using a step-up dosing regimen until predefined discontinuation criteria were met. Overall, 68 patients were enrolled in the dose-escalation phase; 11 (16.2%) experienced DLTs, and all patients experienced at least one TRAE, while 64 (94.1%) developed at least one grade ≥3 TEAE. Despite transient reductions in disease burden observed in some patients, no objective responses were reported [79].

More encouraging results have been reported with AMG 330, a CD33 × CD3 bispecific T-cell engager. In a phase I study including 77 patients with R/R AML treated at doses ranging from 0.5 μg/day to 1.6 mg/day in 14- or 28-day cycles, the maximum tolerated dose was not reached, and the median treatment duration was 29 days. The most frequent TRAEs were CRS (78% of patients, 10% grade ≥ 3) and rash (30%). CRS incidence was mitigated through stepwise dosing, prophylactic dexamethasone, and early intervention with tocilizumab. Regarding efficacy, among 60 evaluable patients, 8 achieved complete remission (CR) or morphologic leukemia-free state [80].

AMG 673 is a novel half-life-extended BiTE targeting CD33 and CD3. In heavily pretreated R/R AML patients, a reduction in blast counts was observed in 12 of 27 (44%) evaluable patients, including 6 patients with ≥50% reduction from baseline. One patient treated at 36 μg achieved CRi, accompanied by an 85% reduction in BM blasts. Across all treated patients, the most common TRAE was CRS, reported in 15 of 30 patients (50%), including grade 1 (n = 6), grade 2 (n = 5), and grade 3 (n = 4), with no grade 4 events. Serious TRAEs occurred in 37% of patients, and 50% experienced grade ≥3 TRAEs, including hepatic enzyme elevations, CRS, leukopenia, thrombocytopenia, and febrile neutropenia [81].

AMV564 is a novel bivalent (2:2) CD33 × CD3 bispecific T-cell engager that has shown encouraging early clinical activity. In a phase I study, 36 patients were enrolled across 10 dose cohorts (0.5–300 μg/day), most of whom had adverse molecular or cytogenetic risk features. No DLTs, grade ≥3 CRS, or CRS-related deaths were observed. The most common grade ≥3 TRAE was anemia (11%). Regarding efficacy, reductions in BM blasts were observed in 17 patients (49%), including one CR during cycle 1 at 200 μg/day, one CRi during cycle 2 at 150 μg/day, and one partial response during cycle 1 at 100 μg/day [82].

#### 1.4.3. Additional Targets and Emerging Approaches

Preclinical and translational studies have identified CLEC12A (C-type lectin domain family 12 member A; also known as CLL-1 or MICL) as a promising alternative target for immunotherapy. CLEC12A is implicated in the leukemogenic process of NUP98::NSD1 AML [83]. Given that a subset of CD33-negative AML cases expresses CLEC12A, a novel tri-specific killer engager (TriKE) targeting CD16–IL-15–CLEC12A has been developed. Preclinical data demonstrate potent NK-cell activation and selective killing of AML blasts while sparing normal HSCs [84,85].

Other approaches have explored NK-cell-mediated cytotoxicity using CD16–IL-15–CD33 TriKE constructs. The first-in-human study of GTB-3550, administered via continuous infusion, demonstrated NK-cell expansion without clinically significant target-mediated toxicity. However, no objective clinical responses were observed at the initial low-dose cohorts, although immune activation provided proof-of-mechanism in humans. Ongoing dose escalation will determine whether NK-cell proliferation translates into clinical efficacy. GTB-3650 was subsequently evaluated in four patients with R/R AML with adverse biological features (two treated at 1.25 μg/kg/day and two at 2.5 μg/kg/day). At the end of two cycles, two patients achieved stable disease and two experienced disease progression. No TRAEs, CRS, or DLTs were reported, and dose escalation to 5 μg/kg/day is ongoing [86,87].

AMG 427 is a CD3 × FLT3 (CD135) bispecific antibody that has demonstrated potent T-cell-dependent cytotoxicity against AML blasts in vitro. However, treatment was associated with increased PD-1 expression, suggesting a potential immune escape mechanism [88].

Finally, preclinical studies of ISB1442, a first-in-class CD38 × CD47 bispecific antibody, have shown effective killing of primary AML and T-ALL samples in both heterologous macrophage-based assays and autologous settings, supporting its further clinical development in these hematologic malignancies [89].

Table 1 summarizes ongoing and completed clinical trials evaluating bsAbs in human AML, with a focus on target antigens, clinical setting, efficacy signals, and key toxicities.

## 2. Discussion

Nowadays, high-risk AML with adverse molecular or cytogenetic features and R/R AML or high-risk MDS represent an unmet clinical need, with limited efficacy of currently available therapies [6]. Over the last decade, our knowledge of the BM microenvironment and the immune system in hematologic diseases has greatly increased. At the same time, major advances have been made in other neoplasms, including lymphoid malignancies, using mAbs and bispecific antibodies. Several studies have shown that combining immunotherapies with conventional chemotherapies or, more recently, with other biologic agents can enhance the antineoplastic effect by targeting both leukemic cells and the permissive microenvironment that supports their survival and proliferation.

**Figure 3:**
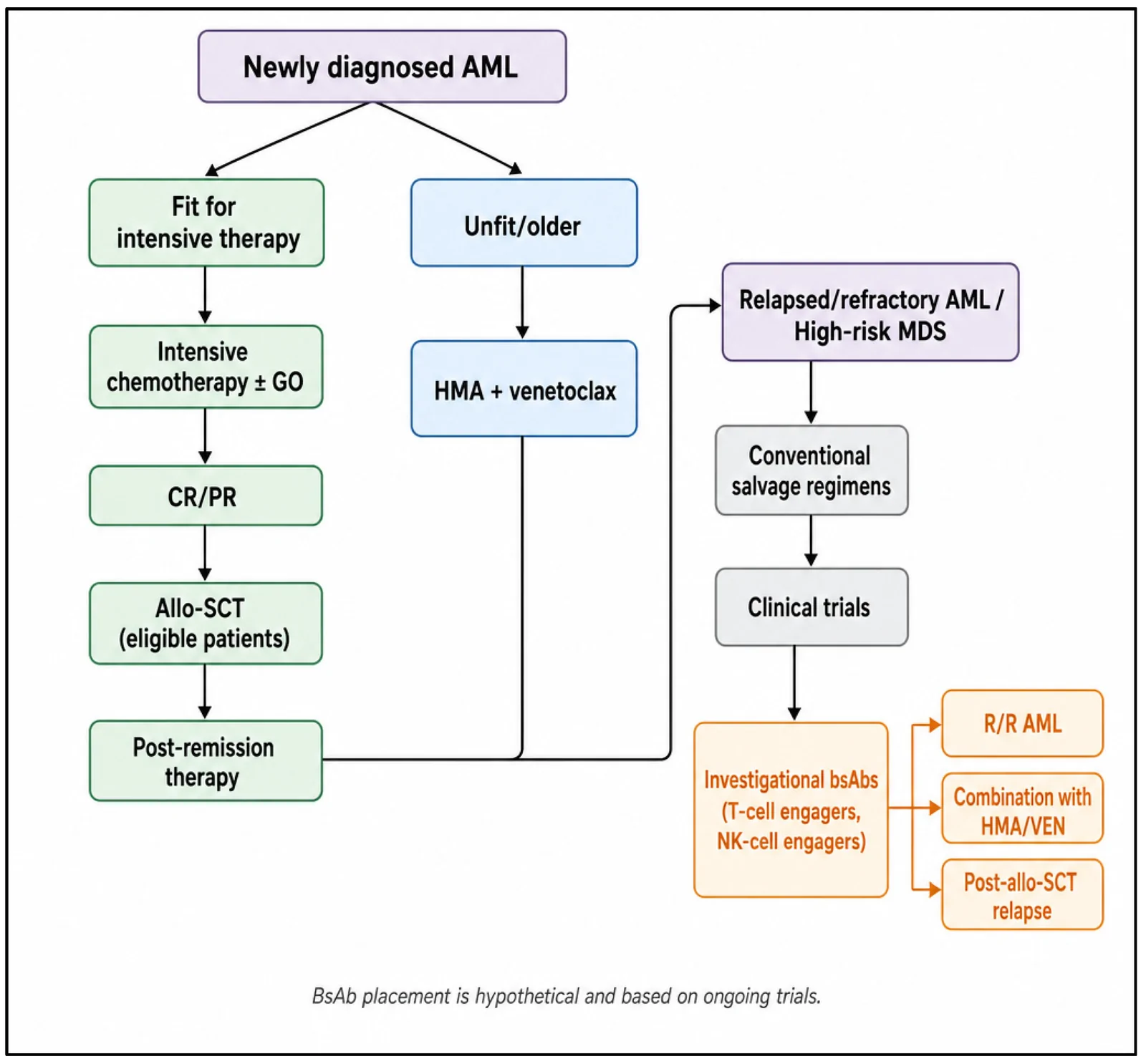
**Potential positioning of bispecific antibodies in AML therapy.** Schematic representation of current treatment pathways for acute myeloid leukemia (AML) and potential investigational positions for bispecific antibodies (bsAbs). Newly diagnosed patients are stratified according to fitness for intensive therapy. Fit patients typically receive intensive chemotherapy, with or without gemtuzumab ozogamicin (GO), followed by complete or partial remission and, when eligible, allogeneic stem cell transplantation (allo-SCT) and post-remission therapy. Unfit or older patients generally receive hypomethylating agents (HMAs) in combination with venetoclax. Disease relapse or refractoriness can occur after either frontline pathway, leading to the relapsed/refractory (R/R) AML or high-risk MDS setting, where conventional salvage regimens and clinical trials are considered. BsAbs are currently being evaluated in clinical trials mainly in R/R AML and high-risk MDS, as monotherapy or in combination with HMA/venetoclax backbones, and are also being explored in the context of post-allo-SCT relapse. The positions shown are hypothetical and reflect ongoing investigational strategies rather than approved indications. Partially prepared with the aid of an AI-based illustration tool and then edited by the authors.

In AML, the role of the immune system has been less clear until recent years, and immunotherapies have only recently been developed. Thus, while in ALL and lymphomas many bispecific antibodies are already in clinical use, all bsAbs in AML are still in early clinical trials and many questions remain unanswered. In this evolving therapeutic landscape, bsAbs are being investigated predominantly in the relapsed/refractory setting and in high-risk myeloid neoplasms, often in combination with established backbones such as HMAs and venetoclax, and in some cases after allo-SCT failure. Figure 3 provides a simplified overview of current AML treatment pathways and highlights potential investigational positions for bsAbs, emphasizing their evaluation mainly in R/R AML and high-risk MDS, as well as in combination and post-transplant strategies.

Available clinical data indicate an acceptable safety profile, although some weaknesses have already been identified. First, the ideal target and the ideal bispecific antibody format have not yet been defined. T-cell engagers are currently the strategy with the most robust efficacy data, showing strong cytotoxic activity in an MHC-independent manner. Unfortunately, their effect depends on lymphocyte count, and some studies have excluded lymphopenic patients from enrollment. Moreover, they are associated with CRS. In recent years, thanks to the experience with bsAbs in other hematologic malignancies and with CAR-T, CRS management has become less daunting. Clinicians have gained confidence in recognizing the early clinical and laboratory signs of CRS, and the use of tocilizumab has become widespread. These advances help explain why, in the aforementioned trials, although CRS rates were high, most events were grade 1 or 2 and were easily managed [61,62,63,64,65,66,67,68,69,70,71,72,73,74,75,76,77,78,79,80,81,82,90,91,92,93].

On the other hand, TriKEs, which exploit NK cell-mediated killing, appear to be an interesting alternative strategy, although clinical data are still very limited, in particular because the rate of CRS seems to be much lower. In addition, NK cells appear to target LSCs effectively, potentially leading to deeper and more prolonged remissions, although currently available data are not yet strong enough to fully support their efficacy [84,85,86,87].

The advent of bsAbs also raises the issue of immune escape and drug resistance. Immune escape in AML has been widely studied, and several mechanisms have been identified: (i) loss of immunogenic stimuli and overexpression of immunomodulatory molecules: (ii) immune cell exhaustion, marked by overexpression of CTLA-4, PD-1, LAG-3, TIM-3, and other checkpoint molecules on T lymphocytes, leading to T-cell anergy; and (iii) secretion of immunomodulatory cytokines by cells in the BM microenvironment, particularly LSCs and MSCs [94].

From this perspective, combination strategies using bsAbs together with small molecules capable of reducing BM microenvironment-mediated immunosuppression could represent a successful approach. Checkpoint inhibitors have shown conflicting results in AML, with better outcomes when combined with other biologic therapies [95,96]. Similarly, TIM-3 inhibitors act by modulating the BM miroenvironment stimulating T-cell activation against AML blasts. These molecules may enhance immune responses when used together with bsAbs, given the ability of TIM-3 inhibitors and checkpoint inhibitors to boost both T-cell and NK-cell activity [97,98]. To date, however, no clinical or preclinical data are available on either of these combination strategies, and further studies are needed to determine whether these combinations are safe or whether simultaneous administration of multiple immune-acting agents increases the risk of CRS.

Ongoing clinical trials are evaluating the efficacy of combining bsAbs with other biologic agents. For example, the clinical trial NCT06634394 is assessing the triplet of APVO436, venetoclax, and azacitidine in newly diagnosed AML patients.

Another open question concerns the role of chimeric antigen receptor (CAR) T-cell therapy in AML. Researchers have shown that developing AML-targeted CAR-T therapy is challenging because of the heterogeneity of target antigen expression among patients, the aforementioned immunosuppressive microenvironment, and the difficulty in avoiding on-/off-tumor toxicity. As for mAbs and bsAbs, multiple targets, including CD33, CD123, CLL-1, NKG2D, and CD7, have been actively explored for CAR-T cells. Although several clinical trials have reported promising results, no CAR-T cell product is yet close to routine clinical use in AML [99,100,101,102,103].

## 3. Conclusions

In recent years, growing knowledge of AML biology and of the BM microenvironment has been changing the treatment paradigm of AML. Many new therapeutic strategies (small molecules, mAbs, bispecific antibodies, and cellular therapies) have been developed, improving remission rates and survival. Many bsAbs have emerged as potential therapeutic strategies in AML. The use of different types of bsAbs targeting distinct cellular antigens has raised the question of which agent represents the optimal therapeutic choice among those currently available. To date, no head-to-head comparisons have been conducted, making it difficult to predict which bsAb will gain greater prominence and shape the future landscape of AML treatment. Among the available agents, bsAbs targeting CD123 have been the most extensively investigated, demonstrating favorable safety profiles and promising efficacy. By contrast, the role of TriKEs, which exploit NK cell-mediated killing, remains to be further elucidated.

In this evolving therapeutic landscape, future studies will be crucial to determine not only which bsAbs will ultimately emerge as the most clinically relevant, but also how they can be optimally integrated into AML treatment algorithms. Key questions include identifying the patient populations most likely to benefit, including those with adverse biological features, who have already demonstrated promising responses [76,77,78], and defining the most effective combination strategies with bsAbs.

## Figures and Tables

**Table 1:** Clinical trials exploring the safety and efficacy of bispecific antibodies in human AML.

Molecule	Target	Format	Clinical Setting	Results	Reference
*bsAbs targeting CD123*					
APVO436	CD123 × CD3	IgG-like bsAb	R/R AML/MDS	ORR 21.7% CRS manageable	[72]
Vibecotamab (XmAb14045)	CD123 × CD3	IgG-like bsAb	R/R AML	ORR ~9%; CRS frequent (mostly ≤ G2); step-up dosing reduces CRS	[74]
Flotetuzumab (MGD006/S80880)	CD123 × CD3	DART	R/R AML	ORR ~30%; CR/CRh 26.7%; activity also in TP53-mutated AML	[76,77]
JNJ-63709178	CD123 × CD3	IgG-like bsAb	AML	High toxicity (CRS, TEAEs); limited efficacy	[78]
*bsAbs targeting CD33*					
JNJ-67571244	CD33 × CD3	IgG-like bsAb	R/R AML/MDS	High toxicity, no objective responses	[79]
AMG 330	CD33 × CD3	BiTE	R/R AML	CR/MLFS in ~8 pts; CRS common (78%)	[80]
AMG 673	CD33 × CD3	Half-life extended BiTE	R/R AML	Blast reduction in 44%; 1 CRi; CRS in 50%	[81]
AMV564	CD33 × CD3	Tetravalent bsAb	R/R AML	49% blast reduction; 1 CR, 1 CRi, 1 PR; no CRS ≥ G3	[82]
GTB-3550	CD16 × IL-15 × CD33 (TriKE)	TriKE	R/R AML	No responses in early cohorts	[86]
GTB-3650	CD16 × IL-15 × CLEC12A	TriKE	R/R AML	SD in 2/4 pts; no CRS or DLTs	[87]

## Data Availability

No new data were created or analyzed in this study. Data sharing is not applicable to this article.
